# Why Doesn’t Primary Biliary Cholangitis Respond to Immunosuppressive Medications?

**DOI:** 10.1007/s11901-017-0345-y

**Published:** 2017-04-27

**Authors:** Antonio Molinaro, Hanns-Ulrich Marschall

**Affiliations:** Department of Molecular and Clinical Medicine, Institute of Medicine, University of Gothenburg, Sahlgrenska Academy, SE-413 45 Gothenburg, Sweden

**Keywords:** PBC, Immunosuppression, Bicarbonate, Cholestasis, AMA, OCA, UDCA

## Abstract

**Purpose of Review:**

The purpose of this review is to discuss reasons why immunosuppressive therapy so far failed in Primary Biliry Cholangitis.

**Recent Findings:**

Even targeted immunosuppressive therapy seems ineffective or potentially harmful.

**Summary:**

Bile acid-mediated cholangiocyte damage, facilitated by insufficient bicarbonate secretion, seems to attenuate the anti-inflammatory and anti-fibrotic actions of immunosuppressant and immunomodulatory drugs in a clinically significant way.

## Introduction

Primary biliary cholangitis (PBC; formerly called primary biliary cirrhosis [1]) is a chronic cholestatic liver disease, characterized by destruction of small intrahepatic bile ducts, leading to fibrosis and potentially cirrhosis with resulting complications [2]. The diagnosis can be made on the basis of two of three criteria: the presence of biochemical evidence of cholestasis based mainly on alkaline phosphatase elevation (ALP >2× ULN, γGT >5× ULN for more than 6 months), presence of anti-mitochondrial antibodies (AMA), and histologic evidence of non-suppurative destructive cholangitis and destruction of interlobular bile ducts [3]. A liver biopsy is not essential in patients with ALP elevation and AMA, but may be required for the diagnosis of concurrent features of autoimmune hepatitis and disease stage [4].

### PBC Is an Immune-Mediated Disease

Primary biliary cirrhosis (PBC) is considered a prototypical autoimmune disease because of the well-defined nature of the autoantigen, its homogeneous clinical expression, and its overwhelming predominance in females. PBC is characterized by production of a multilineage immune response to mitochondrial autoantigens that is directed at the E2 component of the 2-oxo-dehydrogenase pathway, particularly PDC-E2 [5]. Biliary epithelial cells are targeted in PBC and express T cell ligands that are thought to be essential for the induction of biliary epithelial autolysis. The highly complex mechanisms for selective biliary cell destruction and potential unique pathways for therapeutic interventions have recently been reviewed [5••^6^, ^7^].

Established risk factors for PBC are age, gender, and ethnicity. Five genome-wide association studies (GWAS) and two immunochip studies in PBC confirmed associations at the human leukocyte antigen (HLA) locus as in other autoimmune diseases. In addition, a total of 33 non-HLA risk loci were identified including targetable pathogenic pathways involving myeloid cell differentiation, antigen presentation, T cell differentiation, and B cell function in PBC [8–15•, 16]. The role of epigenetics in PBC, e.g., different regulation of gene expression through DNA methylation, is currently under intense investigation [17].

Across years, several animal models of PBC have been proposed aiming to understand the pathophysiology and to develop potential therapeutics in PBC. Only recently, an animal model has been established that reassembles some of the major PBC features such as female gender predominance, alteration of bile acid profiles, production of AMA, and portal ductal inflammation. This mouse model is characterized by chronic interferon-γ overexpression [18], which once again underlines the importance of the immune system in PBC pathogenesis.

### Role of Gut Microbiota and Apoptosis in PBC Pathogenesis

In addition, the gut microbiota has been identified as an important factor in the PBC pathogenesis [5••]. Direct evidence comes from metagenomic studies that show a different microbiota composition in subjects with PBC compared to healthy controls [19]. Moreover, it has been shown that subjects with PBC have an altered intestinal barrier as it is also found in celiac disease, which shares some common immunological features with PBC [20]. Several clinical reports have also documented an increased incidence of celiac disease in PBC patients [20]. Furthermore, sera from patients with PBC can cross-react with mucosal antigens, and immune responses against intestinal microbes may be promoted by the finding of increased intestinal permeability and defective barrier function in PBC. This has led to the suggestion that PBC may also be triggered by exposure to enterobacterial antigens [21].

One of the most important functions of gut microbiota is to metabolize bile acids produced by the liver [22], which at least in experimental animal models substantially impacts on the activation of the farnesoid X receptor (FXR) [23•], which is a nuclear bile acid receptor predominantly expressed in the intestine and in the liver [23•]. Intestinal activation of FXR leads to the production of fibroblast growth factor 19 (FGF19), which after binding to the FGF receptor 4/beta klotho heterodimer triggers a signaling cascade that represses CYP7A1, which is the key enzyme of bile acid synthesis [23•]. In addition, bile acid activation of FXR in the liver inhibits bile acid synthesis and uptake and increases their excretion, which protects against toxic bile acid injury and enhances liver regeneration in animal models of cholestasis. Thus, modulation of the bile acids-FXR-FGF19 pathway is one of the most promising therapeutic options in PBC [24].

Increased cholangiocyte apoptosis is one of the most important histological features of PBC [25, 26]. The apoptotic cell damage is secondary to the activation of autoreactive cells targeting biliary epithelial cells [27]. Not only the loss of immunological tolerance mediated by type 1 T helper lymphocytes but also altered immunological function of cholangiocytes plays an important role in the increased apoptosis in PBC. Cholangiocytes themselves are involved in maintaining the immunological stimuli during PBC in different ways [28]. Apoptotic cholangiocytes can continuously release intact self-antigens, which is secondary to the lack of antigen modification after apoptosis (glutathionylation of the PDC-E2 antigen) [29]. Moreover, in PBC, cholangiocytes are able to act as antigen-presenting cells [30].

In the cholangiocyte apoptosis complex scenario, other non-cell-mediated pro-apoptotic signals are involved. Hydrophobic bile acids or reactive oxygen species can be responsible for the cholangiocyte damages that lead to apoptosis [28].

Fibrosis in PBC is the consequence of a continuous cholangiocyte proliferation after apoptotic damage. This mechanism leads to a reduction in the functional bile ducts and to the clinical features of advanced PBC with fibrosis and ultimately cirrhosis [2]. Tumor necrosis factor α (TNF-α) is one of the most important inflammatory modulators that can induce pro-apoptotic signals in PBC [31]. Recent evidence has shown that the TNF-related apoptosis-inducing ligand (TRAIL) could be a target pathway for the onset of PBC and of cholestatic diseases in general [32, 33].

### Approved Treatment of PBC

Until recently, only ursodeoxycholic acid (UDCA) had been approved for the treatment of PBC by American and European drug agencies and was recommended as first-line treatment by American and European liver associations [3, 4]. Normalization of ALP, or decline by more than 40%, by treatment with UDCA is seen in about 60% of PBC patients and indicates improved outcome [34]. ALP levels are also implemented in recent advanced prognostic algorithms for PBC [35•, 36•]. A number of properties and mechanisms of actions of UDCA have been described, including anti-inflammatory and anti-apoptotic actions, stimulation of secretion of a bicarbonate-rich fluid from cholangiocytes, and others. The relative contribution to the beneficial effect in PBC may depend on the degree of cholestasis [4, 37, 38]. Based on recent phase II and III studies that showed significant improvements of ALP [39, 40••], obeticholic acid (OCA) in 2016 became the second approved treatment for PBC by American and European drug agencies. OCA is a high-affinity ligand of the nuclear hormone receptor FXR. Fibrates alone or in combination with UDCA have been shown to improve biochemistry and, thus, prognostic indices [41, 42]. However, their long-term benefits are controversial. Likewise, the treatment with budesonide in PBC has been controversial, and it might be reserved to non-cirrhotic patients with overlap between PBC and autoimmune hepatitis (AIH) [4]. Of note, the first 1-year pilot trial with prednisolone showed improved serum liver tests and histology but at the expense of markedly increased bone loss [43]. A following study combining prednisolone with UDCA showed histological improvement in PBC in the early stage but was not superior to UDCA monotherapy in terms of liver function tests [44].

### Unsuccessful Treatment Approaches in PBC

Numerous other drugs have been tested in PBC during the last four decades, including anti-fibrotic agents (colchicine, penicillamine, malotilate), antivirals (lamivudine w/o zidovudine), silymarin, sulindac, statins, thalidomide, tamoxifen, and, in particular, immunosuppressants (azathioprine, chlorambucil, cyclosporine, methotrexate, mycophenolate mofetil). These were either only marginally effective, ineffective, or potentially harmful and thus are not currently recommended [3–5••, 45].

The extensive failure of any immunosuppressant and immunomodulatory treatment is paradoxical but was also seen in two recent studies with a more specific immunological approach. Clinical efficacy was either very limited or absent in pilot trials with rituximab [46] and with ustekinumab [47], respectively. Rituximab is an anti-CD20 monoclonal antibody that produces selective B cell depletion and potentially could ameliorate autoimmune disease by decreasing autoantibody production and antigen presentation by B cells. In those six patients who were treated with rituximab, multiple mechanisms were identified by which B cell depletion might lead to clinical improvement in PBC. Of note, significant reduction in ALP was observed that persisted for up to 36 weeks after treatment [46]. In contrast, no overall ALP response or remission was observed at week 12 in those 20 PBC patients who were treated with ustekinumab, which is a human monoclonal antibody that specifically binds to the shared p40 protein subunit of human IL-12 and IL-23 [47]. Interestingly, in those PBC patients who showed a decline in ALP levels during ustekinumab treatment, a modulation of biochemical pathways was observed, including Th17 lymphocytes [47]. A potential benefit of anti-IL-12 treatment had been suggested by increased serum IL-23 levels in patients with PBC [48] and, in particular, by significant genetic associations of PBC for IL-12A and IL-12 receptor beta subunit from GWAS data [8, 16]. The difficulties in translation of GWAS findings into a successful drug might be explained by the fact that non-HLA genetic findings are likely to represent general regulatory aspects of disease susceptibility that interact with certain environment factors to drive inflammation rather than specific factors that are involved in biliary disease development [49].

End-stage PBC can only be treated with liver transplantation resulting in graft and patient survival of, e.g. 77 and 82%, respectively, at 5 years in the Nordic countries [50]. Despite these good results in terms of survival due to improvement in surgical techniques and new immunosuppressive drugs, recurrence of PBC after liver transplantation (rPBC) is diagnosed in up to 29% of patients at 10 years after transplantation [51]. Several factors have been involved in the pathogenesis of rPBC such as donor-recipient gender or HLA-DR locus mismatch, young age at transplantation, older donor age, IL-12 polymorphisms, severe cholestasis, very high IgM, and others (recently reviewed in [51]). Treatment with cyclosporine compared to tacrolimus showed a reduced incidence of recurrence of PBC after liver transplantation [52]. The mechanism underlying the protective effect of cyclosporine is still unknown, but some reports suggest a protective role of cyclophilins towards viral damage to the biliary epithelia after transplantation [51]. Few data are available on the outcome of rPBC. A retrospective analysis of 154 PBC patients did not find rPBC to be associated with death or liver retransplantation [53]. Of note, UDCA therapy did not impact on graft or patient survival in rPBC [53]. The liver transplant experience in PBC and the common development of rPBC again points to PBC as an unusual immune-mediated liver disease as even posttransplant immunosuppressive therapy is unable to prevent disease recurrence.

### Why Immunosuppressive Agents Might Have Failed in Treatment of PBC

A key feature of PBC is cholestasis that in early PBC cannot be exclusively attributed to the loss of bile ducts since serum markers of cholestasis are already elevated before the onset of significant ductopenia. Thus, a certain functional component is suggested [54]. The biliary bicarbonate “umbrella” hypothesis might give us an explanation [38, 55, 56]. Cholangiocytes secrete large amounts of bicarbonate via the Cl^−^/HCO_3_^−^ anion exchanger 2 (AE2, also known as SLC4A2). Patients with PBC were found to have decreased gene expression levels of AE2 in the liver [57]. This has been linked to increased expression of miRNA-506 in PBC cholangiocytes, which targets the mRNAs of AE2 [58] and type III inositol 1,4,5-trisphosphate receptor [59], both posttranscriptional inhibitions resulting in impaired biliary secretion. Furthermore, AE2-deficient mice develop AMA specific against PDC-E2 and other immunological features resembling PBC [60, 61]. Sufficient excretion of HCO_3_^−^ via AE2 is needed to maintain the physiological biliary pH > 7.4, in order to minimize the abundance of protonated glycine-conjugated bile salts (p*K*a around 4) that otherwise might enter the cholangiocytes as free acids; HCO_3_^−^ thus provides a cellular damage preventing “umbrella” [55]. In addition to this alkaline barrier, an intact glycocalyx, by stabilizing the alkaline pH microclimate close to the apical membrane, helps to prevent the diffusion of toxic monomeric bile salts into the cell where they induce apoptosis [56]. Bile salt-induced apoptosis in cholangiocytes was found to be stimulated by enhanced soluble adenylyl cyclase activity due to increased intracellular HCO_3_^−^ in consequence of reduced AE2 expression, together with bile salt-triggered release of Ca^++^ from the endoplasmic reticulum [62]. Altogether, changes related to impaired function of AE2 may render cholangiocytes more immunogenetic, including the aberrant expression of PDC-E2, and susceptible to autoimmune responses. The damaged plasma membrane then might activate B and T cell-mediated immune responses. At this stage, bile acid-mediated cellular damage might have gone so far that immunosuppressants and immunomodulators no longer can reverse the ongoing inflammation and development of fibrosis and cirrhosis.

## Conclusion

PBC as a cholestatic autoimmune disease most likely needs two different therapeutic approaches, one that improves bile flow and biliary secretion and one that targets autoimmunity. Immunosuppressive therapy in PBC in the future may have a role in a disease stage-based approach aiming to reconstitute tolerance by attenuation effector T cell activation, deployment of natural immune checkpoints, or reconstitution of regulatory T cell function [7].
